# Secreted molecules as modulators of somatic embryogenesis efficiency - an overview

**DOI:** 10.3389/fpls.2026.1847514

**Published:** 2026-06-26

**Authors:** Rita Pires, Fátima Milhano Santos, Lénia Rodrigues, Augusto Peixe, Hélia Cardoso

**Affiliations:** 1 MED Mediterranean Institute for Agriculture, Environment and Development & CHANGE Global Change and Sustainability Institute, Institute for Advanced Studies and Research, Universidade de Évora, Évora, Portugal; 2 Health Research Institute-Fundación Jiménez Díaz University Hospital, Universidad Autónoma de Madrid (IIS-FJD, UAM), Madrid, Spain; 3 MED Mediterranean Institute for Agriculture, Environment and Development & CHANGE Global Change and Sustainability Institute, Departamento de Fitotecnia, Escola de Ciências e Tecnologia, Universidade de Évora, Évora, Portugal; 4 MED Mediterranean Institute for Agriculture, Environment and Development & CHANGE Global Change and Sustainability Institute, Departamento de Biologia, Escola de Ciências e Tecnologia, Universidade de Évora, Évora, Portugal

**Keywords:** cell reprogramming, extracellular vesicles, gene expression, metabolome, nurse cell cultures, secretome, conditioned medium, somatic embryogenesis efficiency

## Abstract

Somatic embryogenesis (SE) is a biological process in which somatic cells undergo molecular reprogramming, leading to embryo formation. SE is considered the most efficient plant propagation system, supporting breeding programs that use genetic engineering/editing and omics-based research. However, its success depends on a complex interaction between internal and external factors, including biomolecules released by explants into the culture medium. The use of nurse cell cultures, established decades ago, exemplifies this process. Nevertheless, few studies have characterised the secreted molecules involved or investigated their role in SE efficiency. This review compiles current knowledge on molecules released during SE, focusing on secreted proteins and metabolites. In addition, the involvement of extracellular vesicles (EVs) as modulators of SE response is hypothesised, emphasising their capacity to mediate targeted communication during the early stages of embryogenic reprogramming. EVs transport regulatory biomolecules and may participate in coordinating the cellular responses triggered by SE-inducing stimuli, as supported by the recurrent identification of EVs-associated proteins linked to stress perception and developmental signalling. Understanding how these extracellular factors interface with intracellular pathways could advance efforts to optimise SE and broaden its biotechnological applications.

## Highlight

- Extracellular biomolecules influence cell reprogramming responses, enhancing somatic embryogenesis efficiency for improved plant biotechnology applications.

## 1 Introduction

Somatic embryogenesis (SE) is a morphogenic process driven by complex reprogramming mechanisms that enable cell dedifferentiation, reacquisition of meristematic competence, and the *de novo* differentiation of embryogenic structures (Aguilar-Hernández and Loyola-Vargas, 2018). Regardless of its occurrence under natural conditions, SE has been gaining high practical interest for the large-scale *in vitro* propagation of elite cultivars. It gains particular relevance for clonal propagation of woody plant genotypes that are recalcitrant to conventional methods, such as the semi-hardwood cuttings and the microcuttings (Egertsdotter et al., 2019). It also represents the most promising *in vitro* regeneration system to support molecular breeding programs, owing to its high efficiency in regenerating whole plants from single cells (Bogdanović et al., 2021).

According to the theory of plant cell totipotency, any living plant cell that maintains its integrity can undergo a complex reprogramming process involving molecular, biochemical, and physiological changes, ultimately leading to the development of a new plant (Nic-Can et al., 2015). Despite this broad definition of totipotency, it is well known that not all explants respond similarly to embryogenic stimulus, even when taken from the same plant (Fehér, 2019). The developmental and physiological status of the explant tissue are key factors that define the cell’s ability to respond efficiently to SE stimuli. This is particularly evident in woody species, where SE efficiency declines with tissue ageing (Cardoso et al., 2010; Nic-Can and Loyola-Vargas, 2016; Hazubska-Przybył et al., 2020). Explant age has thus been recognised as a major limiting factor in the success of regeneration systems involving *de novo* morphogenesis (Nhut et al., 2023; Zhang X. et al., 2025). Protocols based on SE induction, established for woody plant species, report mainly the use of tissues taken from zygotic embryos (Corredoira et al., 2013; Kim, 2015; Pires et al., 2020, 2025a) or tissues involved in the development of micro or macrogametophytes (Cardoso et al., 2010, 2019). In comparison with older tissues, juvenile tissues contain a higher number of meristematic cells contributing to efficient cell response upon the SE stimulus (Yang and Zhang, 2010).

Moreover, several events control this morphogenic process, which is initiated by an SE stimulus perceived by plant cells as a stressful condition (Arnholdt-Schmitt et al., 2016). After stimulus, plant cells immediately activate several signalling cascades that lead to changes in gene expression patterns responsible for the molecular reprogramming events, essential for the reacquisition of cell meristematic characteristics and expression of totipotency (Elhiti et al., 2022). According to Leljak-Levanic et al. (2011), most of the genes expressed during SE encode proteins belonging to four different categories: a) transcription factors; b) proteins acting in the cell cycle; c) enzymes involved in the biosynthesis of growth regulators, most notably auxins; and d) proteins involved in signalling pathways. More recently, epigenetic mechanisms acting as regulators of gene expression have also been highlighted, with particular emphasis on DNA methylation (Cordeiro et al., 2023; Peng et al., 2025) and miRNA (Ferraz et al., 2023; Cordeiro et al., 2025).

Although extensive research has elucidated the molecular mechanisms underlying SE and its efficiency, most studies have focused on plant tissue, and only a few have considered the role of the extracellular environment. At the tissue level, the apoplast – comprising the continuum external to the plasma membrane, including cell walls and intercellular spaces – plays a fundamental role in maintaining cellular integrity and function (Wang et al., 2018). The apoplastic fluid (APF), which circulates within this compartment (Tanveer et al., 2014), is a key mediator of multiple physiological processes, including the passive transport of water, ions, and nutrients, thereby facilitating mass flow across tissues, as well as plant defence responses and intercellular signalling involved in stress adaptation (Nielsen and Thordal-Christensen, 2013; Karimi et al., 2025). Establishing a parallel between the whole-plant system and *in vitro* liquid cell culture systems, the culture medium can be considered a substitute for the APF, functioning as a surrogate extracellular environment. In this context, the culture medium accumulates biomolecules secreted by cells, including a diverse array of proteins, metabolites, and nucleic acids (such as miRNAs and siRNAs) released through different secretory mechanisms, as well as extracellular vesicles (EVs), which transport these and other bioactive molecules involved in intercellular communication (Woith et al., 2021). This culture medium containing secreted biomolecules is referred to by several authors as conditioned medium (CM) (Umehara et al., 2005; Nic-Can et al., 2015).

The role of CM in modulating SE efficiency has been recognised since the 1980s, when nurse culture systems were introduced to promote the formation of somatic embryos in recalcitrant tissues (Eigel and Koop, 1989). Several studies have aimed to identify the medium components responsible for modulating SE responses and promoting growth, mostly focusing on proteomics and metabolomics approaches. Extracellular proteins, collectively referred to as the secretome, have been reported to regulate several biological and physiological processes, including signalling, cell proliferation, responses to stress conditions, and cell–cell interactions (Tanveer et al., 2014), which therefore could function as promoters or modulators of SE (Duchow et al., 2016). Likewise, several extracellular metabolites have been identified as positive or negative regulators of SE responses (Nic-Can et al., 2015). Regarding the EVs, besides its identification in liquid cell cultures, its potential involvement in morphogenic processes in plants remains largely unexplored. In mammals, EVs have been shown to play crucial roles in physiological processes and cell-to-cell communication, and they can even induce secondary embryonic development by regulating organogenesis (Krause et al., 2018; Mulzer et al., 2025). Consequently, vesiculomics, the comprehensive study of EVs, including their biogenesis, molecular composition, functions and roles in cell-to-cell communication (Clift et al., 2024; Meng et al., 2024), has emerged as a rapidly expanding field in animal research (Mazzarella et al., 2024). Similar to animal systems, plant-derived vesicles can transport small non-coding RNAs (sRNAs), including small interfering RNAs (siRNAs) and microRNAs (miRNAs), suggesting a regulatory role in gene expression and potential involvement in cell proliferation and differentiation (Cui et al., 2020; Rito et al., 2025). In the context of SE, it can be hypothesised that these EVs may carry key biomolecules that mediate molecular reprogramming, thereby modulating cellular embryogenic competence.

Understanding these secreted factors may contribute to the development of improved plant propagation protocols and open new perspectives for biotechnological applications. This review provides an overview of the current knowledge regarding proteins and metabolites involved in SE that have been identified in the extracellular environment and may act as SE modulators, some of which may be secreted via EVs-mediated pathways. In addition, the hypothesis that EVs are secreted by *in vitro* embryogenic *calli* (EC) and act as modulators of SE efficiency through the transport of bioactive molecules will be explored.

## 2 Mechanisms for secretion of cellular biomolecules

Secretion is central to plant cellular function, enabling the delivery of biomolecules to the extracellular space and supporting intercellular communication, defence, and developmental plasticity (Ten et al., 2025). These pathways mediate the exchange of proteins and metabolites required for cell wall remodelling, signal transmission, and the establishment of a supportive extracellular environment for embryogenic development.

**Figure 1:**
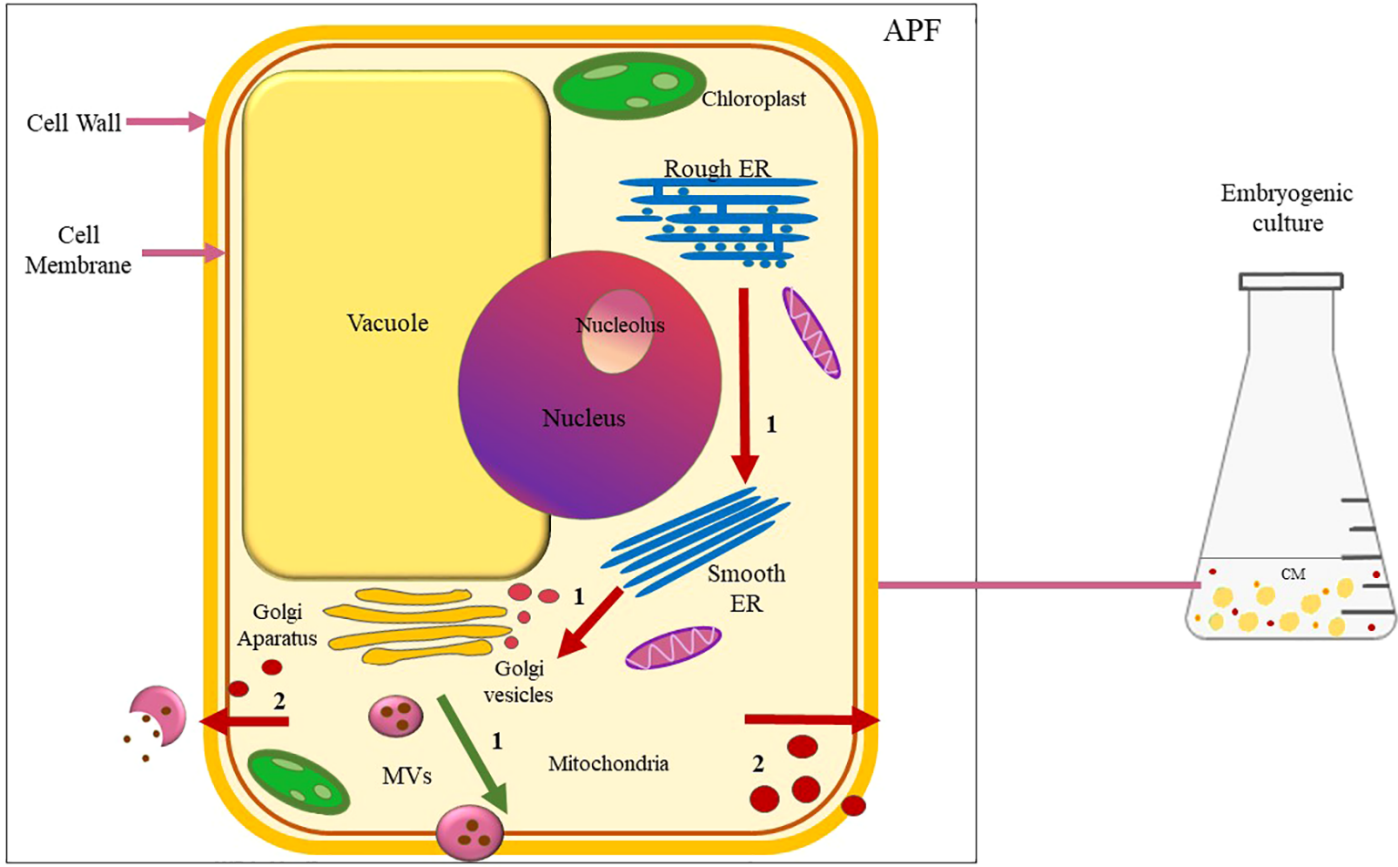
Overview of extracellular proteins secretion pathways by embryogenic cultures into culture medium, following similar procedure described for apoplastic fluid (APF). 1) Conventional secretion pathway through the endoplasmic reticulum (ER) system, Golgi apparatus and by secretor vesicles, which constitute the exocytosis pathway; 2) Non-canonical or unconventional protein secretion (UPS) secretion pathway through direct transfer across the plasma membrane; **(a)** secretion from endosomal/multivesicular body-related components; **(b)** release within plasma membrane-derived extracellular vesicles (EVs) **(c)**.

While the proteome represents the complete set of proteins expressed in a particular cell, tissue, or organ at a given time and under specific conditions (Garrels, 2001), the secretome, in turn, comprises the subset of these proteins that are actively secreted into the extracellular matrix via defined secretory mechanisms (Tanveer et al., 2014). Protein secretion occurs through both the conventional endoplasmic reticulum (ER)–Golgi pathway and the unconventional protein secretion (UPS) pathways, mechanisms recently reviewed by Wang et al. (2018) (**Figure 1**). In the classical pathway, proteins are transported from the ER to the Golgi apparatus, passing through the trans-Golgi network, which sorts them into vesicles directed to the plasma membrane, the cell surface, or intracellular compartments such as endosomes and lysosomes (Wang et al., 2018; Ford et al., 2021). These vesicles subsequently travel through cytoskeletal filaments before fusing with the plasma membrane to release their contents outside the cell. While the conventional pathway predominates, the UPS pathway also contributes to protein export and may be involved in shaping the extracellular environment associated with developmental transitions (Wang et al., 2018; Cui et al., 2020).

The UPS pathway encompasses various mechanisms by which plant cells export biomolecules independently of the classical ER–Golgi route (Wang et al., 2018) (**Figure 1**). These include (i) non-vesicular transport, in which molecules are directly translocated across the plasma membrane via specific transporters or channels; (ii) vesicle-mediated secretion involving intracellular organelles; and (iii) the release of EVs. Plant EVs are lipid-bound structures secreted into the extracellular environment under both physiological and stress conditions (Doyle and Wang, 2019). They carry diverse cargo, including proteins, lipids, carbohydrates, metabolites, and nucleic acids such as small regulatory RNAs, either within their lumen or on their surface, highlighting their role in intercellular communication and long-distance plant defence (Rutter and Innes, 2017; Cui et al., 2020; Zheng et al., 2022). In addition, EVs-associated cargo has been linked to developmental reprogramming processes, suggesting a potential role in modulating the extracellular environment during cellular transitions. Among the proteins commonly enriched in these EVs are tetraspanins (e.g., TET8) (Cui et al., 2020), the secretory syntaxin PEN1 (Liu et al., 2022), and heat shock protein 70 (HSP70) (Ngcala et al., 2020), all associated with EVs biogenesis or stress-related cargo. Proteomic analyses of plant EVs have revealed a diverse set of proteins involved in stress responses, signalling, and cellular remodelling (Chaya et al., 2024), many of which overlap with those reported in the secretome associated with developmental reprogramming processes. In this context, proteins linked to SE-related pathways may also be exported via EVs, suggesting a potential role for these vesicles in shaping the extracellular environment required for embryogenic competence. In addition to proteins, EVs transport diverse metabolites and small RNAs implicated in signalling and developmental regulation (Xiao et al., 2026), further supporting their involvement in modulating the extracellular environment during such transitions. These aspects will be further explored in the following sections.

**Figure 2:**
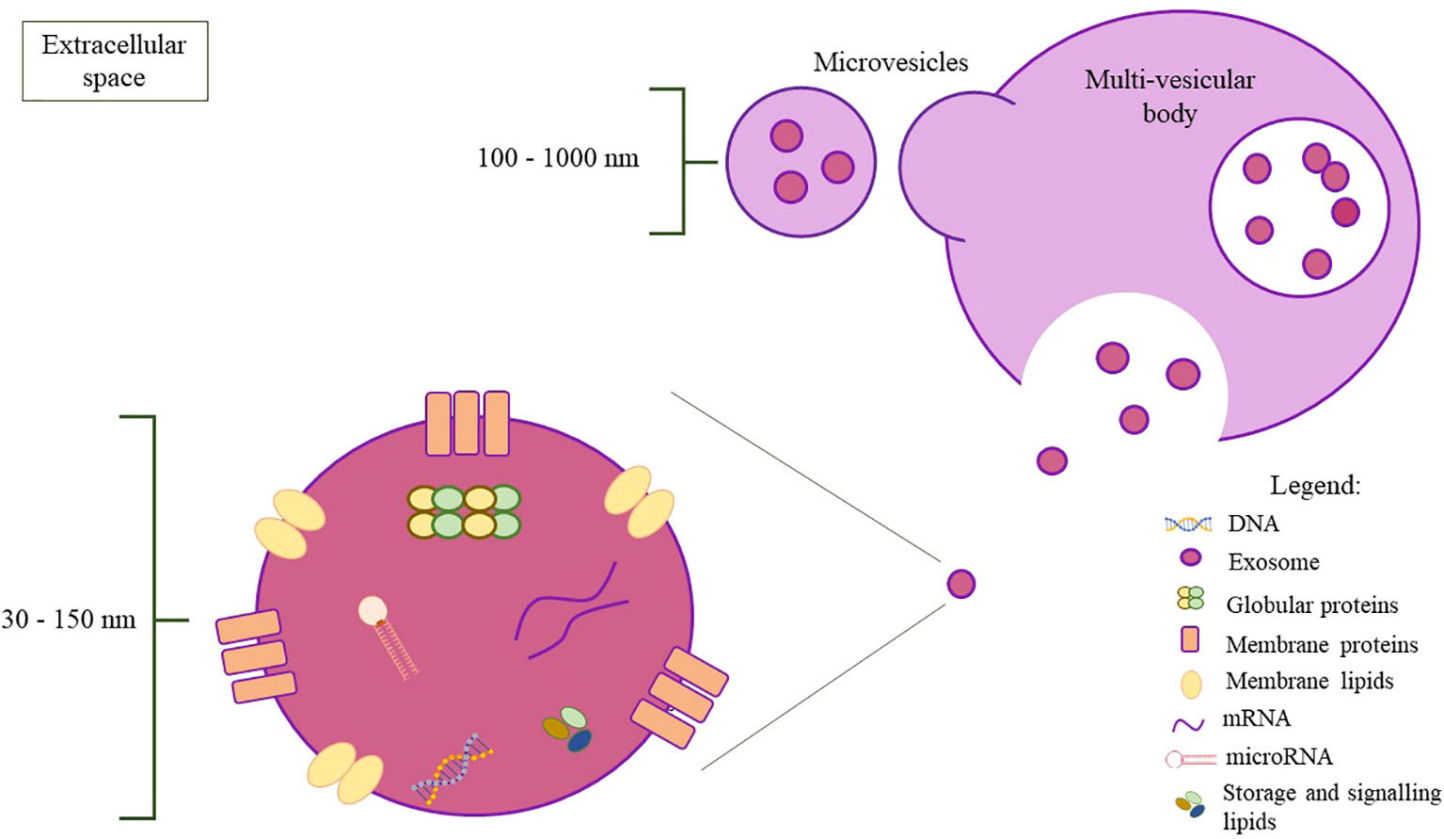
Exosome and microvesicle biogenesis. Extracellular vesicles (EVs) shown include exosomes and microvesicles (MVs). Exosomes are generated within multivesicular bodies (MVBs), which fuse with the plasma membrane to release their intraluminal vesicles.

Plant EVs are generally classified into three main types based on their biogenesis, size, and cargo composition (Chukhchin et al., 2020) (**Figure 2**). Exocyst-positive organelles (EXPOs) are double-membrane compartments, distinct from endosomes and autophagosomes, that mediate the exocytosis of cytosolic material to the cell wall and thus represent vesicle-mediated secretion mechanisms that may contribute to EVs-mediated delivery (Lin et al., 2015). Microvesicles (or ectosomes) are formed by outward budding of the plasma membrane, whereas exosomes (30–150 nm in diameter) are generated within multivesicular bodies (MVBs) and released upon their fusion with the plasma membrane (Liu et al., 2025). Although plant UPS and EVs pathways remain poorly understood, at least three distinct vesicle-mediated secretion mechanisms have been described: (1) EXPO-mediated secretion, (2) outward or inward budding of vesicles, including EVs, from the plasma or endosomal membrane, and (3) fusion of MVBs with the plasma membrane (Cui et al., 2020). EXPO enables the direct transport of cytosolic proteins to the cell wall or extracellular environment (Ding et al., 2014), with the exocyst complex mediating the fusion of post-Golgi vesicles to the plasma membrane (Wang et al., 2018). Exocytosis is integrated with the endomembrane trafficking network, including endocytosis, endosome dynamics, and autophagy (Žárský, 2022), ultimately delivering luminal contents to the apoplast through a two-step process: fusion of the outer membrane with the plasma membrane, followed by degradation of the inner membrane (Ding et al., 2014). Autophagosomes then fuse with the lysosome or vacuole to complete cargo processing (Lin et al., 2015).

The UPS also mediates the extracellular transport of diverse small molecules, particularly secondary metabolites. These may be secreted passively, by diffusion or osmosis when intracellular concentrations are high, or actively via energy-dependent mechanisms. Mauseth (2001) described three types of active secretion: (i) holocrine, involving cell rupture and disintegration; (ii) granulocrine, in which products are stored in vesicles or transported by ATP-dependent processes; and (iii) eccrine, where transporters mediate molecular export across the plasma membrane, also ATP-dependent. Lipids and hydrophobic secondary metabolites are often enriched in exosomes, reflecting their endosomal origin and lipid-rich membranes, which facilitate stable delivery into the extracellular environment, potentially contributing to the setting required for embryogenic competence.

## 3 Involvement of secreted proteins in somatic embryogenesis

Secreted proteins, collectively referred to as the secretome, mediate key processes such as cell–cell communication, cell wall remodelling, and stress signalling, all of which are essential for the induction and progression of SE. Advances in proteomic technologies have enabled the large-scale identification of secreted proteins from the CM of embryogenic cultures. This chapter reviews the methodological approaches used in secretome profiling, highlighting the importance of extraction and sample preparation strategies as key determinants of the subset of proteins ultimately identified. It also examines the analytical methodologies employed, including gel-based and gel-free proteomics, as well as the functional roles of identified secreted proteins in the context of SE.

### 3.1 Protein extraction and sample preparation

Secretome analysis in plant systems presents distinct technical challenges compared to total proteome studies, as it focuses on proteins released into the extracellular space or culture medium, often at low abundance and within complex biological matrices. Within this context, extracellular proteins include both secreted proteins and cell wall-associated proteins, which may range from loosely bound components that freely diffuse into the culture medium to more tightly associated proteins embedded in the cell wall polysaccharide matrix or APF. Consequently, their recovery depends strongly on the extraction strategy employed, which may differentially target loosely or tightly bound protein fractions (Ghahremani et al., 2016). Unlike whole-tissue proteomics, where protein extraction is performed directly from biological material, secretome studies rely on the efficient recovery of extracellular proteins while minimising interference from residual medium components and non-secreted proteins. Low protein abundance, contamination from co-isolated components, and sample loss during preparation can further limit reproducibility and sensitivity in mass spectrometry–based workflows (Mattamana et al., 2025). The protocols described for the recovery of extracellular proteins from culture medium include centrifugal filtration (Charmont et al., 2005; Pires et al., 2025b), tangential flow ultrafiltration (Basu et al., 2006), and freeze-drying (Oh et al., 2005; Tran and Plaxton, 2008). In some cases, an additional concentration step is applied, typically involving solvent precipitation using acetone or methanol (Kaffarnik et al., 2009; Pires et al., 2025b). Additional strategies to improve protein yield and stability have also been reported. For instance, the inclusion of polyvinylpolypyrrolidone (PVPP), which contributes to the removal of phenolic compounds and reduces non-specific interactions that can lead to protein aggregation and precipitation, has been shown to enhance protein recovery and expand proteome coverage (Charmont et al., 2005).

In addition, the release of intracellular proteins into the extracellular environment, due to cell damage or lysis during sample handling, represents a well-recognised limitation in secretome studies. This can compromise the specificity of the extracellular proteome, requiring careful experimental design and data interpretation to distinguish truly secreted proteins from potential contaminants. To assess sample purity, the presence of intracellular marker enzymes, such as malate dehydrogenase (Loureiro et al., 2011; Figueiredo et al., 2021; Pires et al., 2025b), aldolase (Ebner et al., 2016), and glucose-6-phosphate dehydrogenase (Oh et al., 2005), is commonly evaluated using immunoblotting or enzymatic activity assays. In suspension cultures, direct microscopic observation has also been employed to assess cell viability and detect dead or damaged cells that may contribute to secretome contamination (Alexandersson et al., 2013). Overall, careful optimisation of protein extraction and sample preparation is essential to ensure reliable secretome characterisation and to minimise factors that may affect downstream proteomic data analyses.

The recovery of extracellular proteins can be achieved through two main approaches: collection from CM in liquid culture systems or extraction of APF from intact plant tissues. CM-based extraction is a non-destructive strategy in which proteins secreted by cultured cells or tissues accumulate naturally in the surrounding medium, allowing repeated sampling and generally providing sufficient material for downstream analyses (Abbineni et al., 2022). Nevertheless, CM may also contain intracellular proteins released from damaged or senescent cells, particularly in long-term cultures, making cell viability assessment essential (Park et al., 2021). In contrast, APF extraction provides more direct access to the native extracellular space and proteins associated with the cell wall. This approach typically involves vacuum infiltration followed by centrifugation or other mechanical procedures to recover APF contents (Chen et al., 2025). However, because tissue manipulation is required, APF extraction is inherently more invasive and more prone to contamination by cytoplasmic proteins released during processing. Consequently, the reliability of both CM- and APF-based secretome analyses strongly depends on careful sample preparation and rigorous quality control procedures to minimise intracellular contamination and ensure accurate characterisation of extracellular proteins.

### 3.2 Methods used for identification of secreted proteins – the secretome profile

The first analysis in this research field was conducted using classical proteomic methods, including gel-based techniques such as sodium dodecyl sulphate-polyacrylamide gel electrophoresis (SDS-PAGE), isoelectric focusing, and two-dimensional polyacrylamide electrophoresis (2DE) (reviewed by Tchorbadjieva, 2006). Nonetheless, gel-free proteomics methods such as Liquid chromatography coupled to tandem mass spectrometry (LC-MS/MS) have emerged in recent years, demonstrating high applicability on secretome characterisation related to SE (Pernis et al., 2023; Pires et al., 2025b).

#### 3.2.1 Gel-based proteomics techniques

Gel-based proteomics techniques have been widely used to analyse protein expression profiles and characterise the secretome associated with SE. SDS-PAGE is a commonly applied method for separating complex protein mixtures according to their molecular mass, allowing the resolution of proteins ranging approximately from 5 to 250 kDa (Wijethunga and Emenike, 2024). Several studies on SE have employed SDS-PAGE to detect extracellular proteins (see **Table 1**). These analyses revealed distinct protein profiles among the studied species, with many proteins identified as extracellular or associated with the cell wall. Interestingly, some extracellular proteins were not detected in cell wall extracts, highlighting the complexity and specificity of protein localisation within plant cell walls. However, owing to the intrinsic limitations of SDS-PAGE in resolving complex protein mixtures and enabling definitive protein identification, it is commonly complemented by additional techniques, such as 2DE and Western blotting, allowing more comprehensive protein separation and characterisation.

**Table 1:** Analytical techniques for protein identification from conditioned medium (CM) of embryogenic cultures across different plant species.

Protein group	Species	Inoculated material	Proteomic technique	References
Arabinogalactan	*Persea americana*	EC	HPLC	Encina et al., 2024
	*Daucus carota* *Gossypium hirsutum* *Pelargonium sidoides*	Cell culture EC EC	Crossed electrophoresis SDS-PAGE; HPLC Size exclusion chromatography	Kreuger and Van Holst, 1993, Poon et al., 2012, Duchow et al., 2016
	*Pinus caribaea*	EC and NEC	SDS-PAGE, Western blot	Domon et al., 2000
	*Sorghum bicolor*	Shoots from *calli* culture	iTRAQ, LC-MS/MS	Ngcala et al., 2020
	*Pinus caribaea*	EC and NEC	SDS-PAGE, Western blot	Domon et al., 2000
Chitinases	*Dactylis glomerata* *Cucurbita pepo*	Proembryogenic masses EC	SDS-PAGE, 2DE, Isoelectric focusing Immunoblotting	Tchorbadjieva, 2006 Ben-Amar et al., 2010
	*Pinus nigra*	EC	SDS-PAGE, Electrophoresis	Fraterová et al., 2013
Esterases	*Arabidopsis thaliana*	EC	SDS-PAGE; MALDI-TOF	Yugay et al., 2023
	*Dactylis glomerata*	EC and NEC	SDS-PAGE	Tchorbadjieva and Odjakova, 2001
	*Marchantia polymorpha*	Cell culture	SDS-PAGE	Izumi et al., 1995
Germin-like proteins	*Olea europaea*	EC	LC-MS/MS	Pires et al., 2025b
	*Saccharum* spp.	EC and NEC	nanoUPLC/-HDMSE	Heringer et al., 2015
	*Pinus caribaea*	Cell lines	SDS-PAGE, Western blot	Domon et al., 1995
Lipid transfer proteins	*Daucus carota*	Cell culture	SDS-PAGE; RNA Gel Blot	Sterk et al., 1991
	*Hordeum vulare*	Cell culture	Immunoblotting; SDS-PAGE	Mundy and Rogers, 1986
	*Vitis* spp.	Cell culture	SDS-PAGE	Coutos-Thevenot et al., 1992
		Cell culture	SDS-PAGE; HPLC	Coutos-Thevenot et al., 1993

EC (Embryogenic *calli*; NEC (Non embryogenic *calli*); HPLC (High-performance liquid chromatography); iTRAQ (Isobaric tags for relative and absolute quantification); LC (Liquid chromatography); MALDI-TOF (Matrix-Assisted Laser Desorption/Ionization Time-of-Flight); MS/MS (Tandem mass spectrometry); SDS-PAGE (Sodium dodecyl sulfate-polyacrylamide gel electrophoresis.

Among gel-based methods, 2DE remains one of the most widely used techniques for protein separation (see **Table 1**). It is considered a powerful tool for the high-resolution separation and fractionation of complex protein mixtures from biological samples (Magdeldin et al., 2014) and has been extensively applied in SE studies. Nevertheless, several technical limitations have been reported, including issues related to reproducibility, limited detection of low-abundance proteins, and difficulties in analysing highly hydrophobic or extremely large or small proteins (Speda et al., 2017). To overcome variability between gels, the two-dimensional differential in-gel electrophoresis (2DE-DIGE) approach has been developed and applied. However, the requirement for specialised equipment, particularly laser scanners for gel image acquisition, remains a limitation for its broader application (Aguilar-Hernández and Loyola-Vargas, 2018).

#### 3.2.2 Gel-free proteomics techniques

Gel-free proteomics techniques, such as LC–MS/MS-based approaches (including label-free quantification and isobaric labelling strategies such as iTRAQ or Tandem Mass Tag, TMT), offer high sensitivity and accuracy with multiplexing capabilities that allow simultaneous identification and quantification of proteins from multiple samples in a single run. By minimising variation across MS runs, these approaches further improve analytical precision (Tian et al., 2019). However, these approaches also present certain limitations. Labelling-based methods, such as iTRAQ and TMT, while enabling the simultaneous comparison of multiple samples (up to 10), are relatively costly and may be affected by issues such as ratio compression, which can compromise quantitative accuracy. Label-free approaches, while more flexible and cost-effective, are more susceptible to run-to-run variability and missing values. In contrast, labelling-based strategies rely on the incorporation of distinct isotopic or isobaric mass tags, such as iTRAQ, TMT, N,N-dimethyl leucine, deuterium isobaric amine-reactive tags, 10-plex isobaric tags, and sulfoxide-based reagents, to enable multiplexed quantitative analysis (Chen et al., 2021). After labelling, the samples are pooled and analysed by MS, enabling the precise detection of dynamic changes in differential protein expression between samples.

In a study by Ngcala et al. (2020), iTRAQ was applied to analyse *Sorghum bicolor* cell suspension cultures upon heat stress, specifically examining secreted proteins enriched in the CM. This approach enabled the identification of 65 secreted proteins. Notably, 84% of these proteins possessed a predicted signal peptide, suggesting targeting through the classical secretory pathway. Nevertheless, to date, the application of LC-MS/MS-based techniques to protein expression profiling and secretome characterisation in SE has remained limited (**Table 1**).

### 3.3 The involvement of secreted proteins in somatic embryogenesis

Several proteins secreted through conventional pathways into the extracellular space – contributing to the CM composition – have been identified as key modulators of SE, acting as either inducers or inhibitors of signalling molecules. This section focuses on classically secreted proteins, typically exported via the ER-Golgi secretory route, although some may also follow UPS pathways. Notable examples include Arabinogalactan Proteins (AGPs), Chitinases, and Germin-Like Proteins (GLPs), which have been consistently reported as extracellular markers of SE, as summarised in **Table 1**. AGPs constitute a large, diverse family of hydroxyproline-rich glycoproteins characterised by type II arabinogalactans linked to hydroxyproline residues and an N-terminal secretion signal (Leszczuk et al., 2023). Their structural variants include classical AGPs, arabinogalactan peptides, lysine-rich, and chimeric AGPs, which actively participate in extracellular matrix remodelling, signalling, and induction of SE and further development of somatic embryos (Pérez-Pérez et al., 2019) (see **Table 1**). Kreuger and Van Holst (1993) demonstrated that AGPs in CM enhance embryogenic efficiency in *D. carota* by reprogramming non-embryogenic cells and restoring embryogenic competence. Later studies confirmed that exogenous AGP supplementation stimulates somatic embryo differentiation and proliferation. In these studies, AGPs were applied directly within the *in vitro* culture system, typically after *callus* induction (Poon et al., 2012), and during the proliferation and embryogenic expression (Encina et al., 2023) phases. Specifically, AGPs extracted from embryogenic tissues were incorporated into the culture medium, enhancing the embryogenic competence and development of established *callus* cultures. This supports their use as SE inducers, especially in recalcitrant genotypes.

Esterases, hydrolytic enzymes that catalyse the decomposition of the ester bond, serve as specific biomarkers of embryogenic cells. In plant cell cultures, such as *Marchantia polymorpha*, the hydrolysis of acetates into the corresponding alcohols occurs rapidly, presumably due to the secretion of esterases into the culture medium (Izumi et al., 1995). Supporting this, esterase-like proteins were first identified in *Dactylis glomerata* embryogenic suspension cultures, where a 36 kDa acidic esterase was secreted during early stages of SE and associated with initial morphogenic processes (Tchorbadjieva and Odjakova, 2001).

Lipid Transfer Proteins (LTPs) are small, ubiquitous proteins that play essential roles in lipid trafficking and intercellular communication in plants. Initially, LTPs have been found associated with cellular membranes, including those of mitochondria and the ER. However, certain LTP isoforms are targeted to cytosolic compartments. These proteins are synthesised as precursors containing N-terminal extensions with sequence features characteristic of signal peptides, indicating subcellular targeting (Kader, 1996). Early studies in *Hordeum vulgare* identified an LTP, initially annotated as a putative amylase/protease inhibitor, which was later shown to be secreted into the aleurone cell culture medium (Mundy and Rogers, 1986). In *D. carota*, an LTP was detected among extracellular proteins in embryogenic cell cultures and in CM, and its encoding gene was shown to be expressed in embryogenic cultures, including in somatic embryos (Sterk et al., 1991). Similar findings were reported in *Vitis* spp., where 9-kDa LTPs were secreted into the culture medium, suggesting a role in membrane dynamics and signalling (Coutos-Thevenot et al., 1992). Notably, overexpression of *VvLTP1* was associated with the maintenance of embryogenic potential (Maes et al., 1997). Alongside LTPs, Chitinases (endo-type chitinases, EC 3.2.1.14) are glycoside hydrolases that decompose β-1,4-N-acetyl-D-glucosamine linkages in chitin (Zieliński et al., 2021). They likely cleave N-acetyl-D-glucosamine residues within AGPs, contributing to extracellular matrix remodelling (Van Hengel et al., 2002). Both endo-chitinases and β-1,3-D-glucanases are constitutively expressed at low levels and show increased accumulation in embryogenic tissues, particularly during the acquisition of embryogenic competence and subsequent embryo development (Domon et al., 2000).

Alongside the action of LTPs in cell wall dynamics, GLPs, members of the cupin superfamily, are apoplastic glycoproteins implicated in modulating the extracellular environment, notably through manganese binding and hydrogen peroxide (H_2_O_2_) production. The activity of GLPs in the extracellular matrix modulates cell wall properties through oxidative cross-linking, crucial for morphogenetic transitions during SE (Mathieu et al., 2006). Initially identified in *Pinus caribaea* var. hondurensis, GLPs such as GP III, GP103, and GP94 have been proposed as molecular markers for SE (Domon et al., 1995). Their expression is enriched at early SE stages in species of *Larix* and *Saccharum*, implicating them in embryogenic identity establishment (Heringer et al., 2018). Recent secretome analyses of *Olea europaea* embryogenic cultures identified GLPs from subfamilies 1 and 2, including GLP2a, in extracellular protein fractions (Pires et al., 2025b), reinforcing their role as extracellular regulators of embryogenic competence.

Recent advancements in SE regulation have highlighted the role of novel extracellular proteins as potential markers. These emerging proteins offer new insights into the molecular mechanisms underlying SE and could enhance the understanding and efficiency of plant regeneration processes. Alpha-amylase (α-amylase) refers to a family of enzymes characterised by the presence of a predicted secretory signal peptide that cleaves internal alpha 1–4 glycosidic bands of starch and other polysaccharides to produce several products such as glucose and maltose (Avwioroko et al., 2018). In plants, these enzymes catalyse starch and glycogen hydrolysis, playing a key role in starch degradation in cereal grain endosperms, where they are synthesised in secretory tissues and released into the endosperm (Dobrenel et al., 2013). Studies on SE in *Medicago sativa* (Kępczyńska and Zielińska, 2006) and *Musa* spp (Kumaravel et al., 2020) have demonstrated that α-amylase expression is regulated by endogenous gibberellic acid (GA) secreted within the embryo. GA synthesised in the embryo is transported to surrounding tissues, where it enhances α-amylase secretion, promoting starch hydrolysis and improving germination by facilitating starch breakdown, supplying the required energy for the germination process (Kumaravel et al., 2020). Although several studies on SE in plant tissues have reported the presence of α-amylase, few have identified this enzyme in the CM. An example is the work on *D. glomerata* suspension cultures, where the α-amylase (DgAmy1) was identified (Rakleova et al., 2012). The authors reported increased α-amylase activity associated with embryogenic competence and microcluster formation, suggesting that α-amylase activity may serve as a physiological marker of the early phases of SE, including embryogenic competence and early differentiation. In *P. nigra*, higher levels of α-amylase were also reported in the CM of highly embryogenic cultures, leading to increased enzymatic activity and enabling the distinction of cell lines based on their embryogenic capacity (Pernis et al., 2023). Similarly, Pires et al. (2025b) identified OeAmy1 and OeAmy2 in the secretome of high-efficiency embryogenic *O. europaea* cultures. The presence of this protein in the CM, which contains the essential carbon source required for *in vitro* culture, highlights the crucial role of Amy in the SE process.

Another group of extracellular proteins identified as implicated in the regulation of SE are the Peroxidases (PODs). PODs are heme-containing enzymes classified as oxidoreductases, involved in plant stress responses. Among them, Class III PODs (EC:1.11.1.7) are particularly notable as secreted enzymes involved in a wide range of physiological and developmental functions. In *P. nigra*, Pernis et al. (2023) reported the accumulation of POD isoforms in the secretome of highly EC cell lines. Similarly (Ngwenya et al., 2024), in *S. bicolor* and Pires et al. (2025b) in *O. europaea* also reported the identification of PODs in CM of high-efficiency embryogenic lines compared with low-efficiency lines. These studies confirm the potential of extracellular POD enzymes as biomarkers of high embryogenic capacity.

Complementing the oxidative activity of PODs in lignification, Dirigent (DIR) proteins have been identified as mediators of regio- and stereoselective coupling of phenoxy radicals during lignan biosynthesis. Subsequent studies demonstrated that recombinant DIR proteins confer strict regio- and stereospecificity to monolignol free radical coupling, highlighting their essential role in lignification (Burlat et al., 2001), as evidenced by their expression in developing xylem and other lignified tissues (Pei et al., 2023). Beyond lignan and lignin formation, DIR proteins are increasingly recognised as key regulators of plant stress responses and cell wall remodelling (Paniagua et al., 2017). In the context of SE, DIRs have been consistently identified in the secretomes from embryogenic cultures of *P. nigra* (Pernis et al., 2023), *S. bicolor* (Ngwenya et al., 2024), and *O. europaea* (Pires et al., 2025b), suggesting a conserved pattern of secretion across species. DIRs are proposed to contribute to extracellular matrix formation and cell wall reinforcement (Gasper et al., 2016; Pernis et al., 2023), potentially modulating the structural and biochemical environment that facilitates totipotency acquisition. Although the precise functions of DIRs in embryogenic induction remain unclear, their recurrent presence in SE-related secretomes and association with cell wall dynamics highlight their potential role in regulating SE process.

### 3.4 Synthesis: functional integration of secreted proteins in somatic embryogenesis

The identification of extracellular proteins across different species and culture systems highlights common functional patterns that help shape the extracellular environment during SE. Rather than acting independently, these proteins likely operate within coordinated networks that regulate cell wall remodelling, signalling, and redox regulation throughout embryogenic development.

Among the most recurrent proteins detected in embryogenic secretomes are those associated with cell wall modification and extracellular matrix organisation, including AGPs, Chitinases, GLPs, and DIRs. These proteins consistently appear in embryogenic secretomes across phylogenetically diverse species, suggesting conserved mechanisms for establishing a permissive extracellular environment. AGPs function as both structural components and signalling molecules, with their glycosylation patterns potentially encoding developmental information (Poon et al., 2012; Chen et al., 2025). Chitinases and GLPs contribute to polysaccharide processing (Zieliński et al., 2021) and oxidative cross-linking (Mathieu et al., 2006), respectively, while DIRs mediate stereospecific coupling of phenolic compounds during lignin formation (Pernis et al., 2023). Together, these proteins orchestrate cell wall loosening and reinforcement in spatially and temporally defined patterns that accommodate cellular dedifferentiation while maintaining tissue integrity. The recurrent identification of α-amylase, PODs, and LTPs in embryogenic CM suggests that extracellular metabolic adjustments accompany cellular reprogramming. α-Amylase activity reflects carbohydrate mobilisation supporting the energetic demands of embryogenic transitions (Pernis et al., 2023: Pires et al., 2025b), while PODs participate in ROS-mediated signalling and cell wall modification (Chen et al., 2026). LTPs facilitate lipid trafficking and membrane remodelling, processes essential for the extensive cellular reorganisation characteristic of SE (Potocka et al., 2012). The coordinated secretion of these proteins may establish metabolic gradients and redox conditions that promote embryogenic competence while protecting cells from oxidative damage.

Current evidence suggests that the extracellular environment during SE functions as an active regulatory compartment rather than a passive reservoir of secreted molecules. The convergence of cell wall-modifying enzymes, signalling molecules, and metabolic regulators in embryogenic secretomes indicates that successful SE depends on establishing a precisely orchestrated extracellular milieu. Future research should prioritise functional validation of these proteins through gain- and loss-of-function approaches and explore their synergistic interactions to develop evidence-based strategies for enhancing embryogenic efficiency in recalcitrant genotypes.

## 4 Extracellular metabolome – the exometabolome

Secondary metabolism in plants produces a wide range of bioactive compounds derived from primary metabolic pathways. These metabolites play essential roles in plant development and stress response, with profiles shaped by genotype, developmental stage, and environmental or experimental conditions (Mo et al., 2009). The secreted metabolome, or exometabolome, comprises metabolites released into the extracellular space, reflecting active secretion or passive diffusion (Kosina et al., 2018).

Due to limited studies on secreted metabolites during SE, this chapter reviews detection methods and highlights key metabolite families potentially relevant to SE, either via active secretion or exogenous supplementation in culture systems.

### 4.1 Methods for characterising the extracellular metabolome

Several analytical techniques have been employed to identify and quantify metabolites, with the choice of method depending on factors such as metabolite concentration, chemical class, physicochemical properties, and the nature of the biological matrix. Among the most used approaches in metabolomics are nuclear magnetic resonance (NMR) spectroscopy and chromatographic methods like gas chromatography (GC) and liquid chromatography (LC), often coupled to detectors such as MS, ultraviolet (UV), or electrochemical sensors (Seger et al., 2013).

NMR spectroscopy is a non-destructive and highly reproducible technique that enables the simultaneous identification and quantification of multiple small molecules through the detection of specific nuclear responses under magnetic fields (Seger et al., 2013); however, its relatively low sensitivity compared to MS-based approaches may limit the detection of low-abundance compounds. In SE studies, this approach has yielded valuable insights into the metabolic adaptations of embryogenic tissues. For instance, Dowlatabadi et al. (2009) used one-dimensional (1D) ^1^H NMR to analyse the CM of somatic embryo cells of *Picea glauca*, identifying both consumed and secreted metabolites in two different culture medium. Mahmud et al. (2014) applied 1D ^1^H and two-dimensional (2D ^1^H ^13^C) NMR to investigate the relationship between nutrient availability and metabolic adaptation in embryogenic and non-embryogenic *calli i* of *S. officinarum* during SE. More recently, Pires et al. (2025b) utilised ^1^H NMR to profile secreted metabolites in EC of *O. europaea*, identifying compounds in the CM that provided key insights into metabolic pathways maintaining embryogenic competence.

In *Coffea* spp., Nic-Can et al. (2015) compared the CM metabolite profiles of *C. arabica* (a recalcitrant SE-responsive species) and *C. canephora* (highly responsive to SE) using GC-MS and ultra-performance LC coupled with electrospray ionisation tandem MS (UPLC-ESI-MS/MS).

The integration of advanced analytical techniques such as NMR, GC-MS, and LC-MS has significantly enhanced the profiling and quantification of metabolites involved in SE, offering comprehensive insights into the extracellular metabolome and the biochemical pathways regulating this complex developmental process. Despite the availability of these highly sensitive and versatile methods, the most impactful findings to date, regarding metabolite secretion and modulation of embryogenic competence, have predominantly emerged from GC-MS and LC-MS analyses of CM. However, LC-MS approaches may be affected by matrix effects and ion suppression, which can influence quantification accuracy (Zhu et al., 2026). Nonetheless, the application of these techniques directly to extracellular metabolome studies remains relatively limited, highlighting a valuable opportunity for future research to deepen our understanding of the metabolite dynamics underlying SE.

### 4.2 The involvement of extracellular metabolites as modulators of somatic embryogenesis response

A deeper understanding of extracellular metabolites is essential, given their crucial role as modulators of the SE process. This chapter explores major classes of extracellular metabolites, such as amino acids, carbohydrates, phenolic compounds, plant growth regulators, and redox-related molecules, highlighting their specific roles and impact during SE development.

Evidence for the active secretion of amino acids by embryogenic cells into the culture medium remains limited, as secretome studies have predominantly identified other classes of metabolites, with most research focusing on their intracellular roles or exogenous supplementation (Pullman et al., 2015; Daniel et al., 2018). Nevertheless, the presence of amino acids in the extracellular environment may contribute to nitrogen availability and metabolic signalling, given their well-established roles in plant metabolism. In particular, pathways related to amino acid and nucleotide metabolism have been linked to embryo development and the acquisition of embryogenic competence (Guo et al., 2019). Phenolic compounds have been shown to play significant roles in regulating SE, often acting as either promoters or inhibitors depending on their concentration and the plant species involved (Umehara et al., 2005, 2007). Nic-Can et al. (2015) identified several metabolites in the CM of embryogenic cultures of the low-competence species *C. arabica*, including caffeine, chlorogenic acid, hydroxybenzoic acid, and trans-cinnamic acid. Among these, caffeine, although present at the lowest concentration, had the strongest inhibitory effect on SE when applied to *C. canephora* cultures during somatic embryo proliferation. Chlorogenic acid also showed a notable negative impact, indicating that certain phenolic compounds abundant in *C. arabica* CM may suppress embryogenic potential in more competent genotypes. Although most studies have focused on the effects of exogenous application, these findings indicate that phenolic compounds present in the extracellular environment can modulate embryogenic responses. In addition, phenylpropanoid metabolism has been associated with stress responses and cell wall reinforcement, processes that may indirectly support embryo development in *Persea americana* (Olivares-García et al., 2020). Moreover, phenylpropanoid metabolism contributes to the synthesis of lignin and flavonoid precursors, which reinforce the cell wall and enhance stress tolerance, thereby supporting somatic embryo development. Overall, these results highlight the role of phenolic compounds as extracellular signals in the regulation of SE, while emphasising the need for further studies addressing their secretion and function in the culture medium.

Plant growth regulators are naturally biosynthesised compounds that regulate various physiological processes in plants. They are broadly classified into growth promoters, including auxins, gibberellins, and cytokinins, and growth inhibitors, such as abscisic acid (ABA), jasmonates, and ethylene (Kępczyńska and Kępczyński, 2023). In SE, plant growth regulators are key components of the culture medium that control the entire process. Additionally, during the induction of embryogenic competence, the presence of endogenous auxins in combination with cytokinins is necessary for the SE process (Shmakov and Konstantinov, 2020). In a study on *P. glauca* (Dowlatabadi et al., 2009), the metabolome of embryogenic culture medium was analysed, revealing changes in secreted and consumed metabolites associated with cellular organisation. The addition of 2,4-dichlorophenoxyacetic acid (2,4-D) and N^6^-benzyladenine (BA), combined with the absence of ABA, inhibited cellular organisation and apical meristem formation, correlating with specific shifts in amino acids and other metabolic products in the medium. Polyamines, often considered non-classical growth regulators, were investigated by Vuosku et al. (2012) in the CM of *P. sylvestris* embryogenic cell cultures during early growth. The authors observed low levels of secreted polyamines, with putrescine notably accumulating while spermidine declined. This shift was linked to increased putrescine production, likely via the arginine decarboxylase pathway, and reduced spermidine and spermine synthesis.

Regarding the role of carbohydrates, they ensure a steady supply when added to the culture medium, preserving cell osmotic potential, and serving both as energy and carbon sources for various developmental processes (Yaseen et al., 2013). Concerning SE, carbohydrates play a significant role in inducing embryogenic competence, acting as a signalling molecule and regulator of gene expression (Dobrenel et al., 2013). In a study by Pires et al. (2025b) in *O. europaea* CM, the sucrose supplied to the culture medium was hydrolysed into glucose and fructose, while the total concentration of these monosaccharides remained equivalent to the initial sucrose level. This pattern reflects concurrent sucrose hydrolysis in the culture medium, likely mediated by extracellular invertase activity, and uptake of glucose and fructose by the cultured tissues, resulting in a relatively stable concentration of soluble sugars despite ongoing carbon utilisation. The availability and metabolism of these simple sugars are closely associated with the activity of carbohydrate-modifying enzymes involved in central carbon metabolism. During glycolysis, glyceraldehyde 3-phosphate dehydrogenase reduces NAD^+^ to NADH, crucial for energy production. To sustain glycolysis and glucose flux, lactate dehydrogenase (LDH) oxidises NADH back to NAD^+^ (Lees et al., 2020), maintaining redox balance essential for energy, cellular homeostasis, and SE induction. This role has been reported in embryogenic tissues of *Brassica napus* (Ashihara et al., 2008) and *Gossypium* spp (Ge et al., 2025). In the same study, Pires et al. (2025b) reported that a highly embryogenic cell line secreted lactate, 1-methylnicotinamide (MNA), 2,3-butanediol, acetaldehyde, and ethanol. Lactate secretion reflects LDH activity regenerating NAD^+^ under low-oxygen conditions. Extracellular lactate and MNA indicate active NAD^+^ metabolism, essential for cellular homeostasis (Zhang W. et al., 2025). Metabolites like NADH offer valuable insights into SE molecular regulation. NAD^+^ and its precursors maintain cellular metabolism and redox balance, linking glucose flux and NAD^+^ regeneration to embryogenic competence as potential metabolic markers.

## 5 The role of extracellular vesicles in somatic embryogenesis

In animals, EVs are involved in early development, where they mediate intercellular communication by transferring proteins, lipids, and regulatory RNAs. EVs contribute to embryo-maternal signalling, embryo development and viability, and occur in biological fluids such as semen, amniotic fluid and blood (Krause et al., 2018). Recent studies further show that embryonic signals can modulate EVs biogenesis and secretion pathways in endometrial cells, highlighting their functional relevance at the embryo–maternal interface (Guzewska et al., 2023). EVs have also been investigated in embryo culture systems, particularly in CM. However, only a limited number of studies have addressed this topic, highlighting both promising biological roles and significant gaps in current knowledge (Xue et al., 2024). Together, these findings illustrate the importance of EVs-mediated communication during animal embryogenesis.

In plants, EVs similarly transport proteins and small RNAs with signalling potential, but direct evidence for their involvement in morphogenesis or SE remains lacking. A major limitation is that most studies detecting secreted proteins during embryogenic induction rely on apoplastic or culture-medium proteomics, which do not distinguish between conventional secretion and EVs-associated release. Nevertheless, proteins such as annexins, tetraspanins (TETs), and heat shock proteins (HSPs) have been frequently identified in EVs-enriched fractions across plant systems (Cui et al., 2020), which could lead us to hypothesise that EVs may play active roles in cellular signalling, membrane dynamics, and cellular reprogramming, thereby contributing to the modulation of the SE response. **Table 2** summarises the main proteins identified in these unconventional secretion pathways, together with their sources, detection methods and proposed functions. The main achievements regarding the methods used for EVs isolation in plants, along with their potential involvement in SE, are presented below.

**Table 2:** Overview of non-classically secreted and EV-associated proteins identified in plants.

EVs	Specie	System	Analysis	Function	Reference
Annexins	*Arabidopsis thaliana*	*In planta*	SDS-PAGE; Immunoblot	Growth, development, and responses to environmental stresses	Koch et al., 2025
		*In planta*	Immunoblot		Rutter and Innes, 2017
	*Solanum lycopersicum*	*In planta*	SDS-PAGE; LC-MS/MS		De Palma et al., 2020
Glycosylphosphatidylinositol-anchored proteins	*Arabidopsis thaliana*	*In planta*	RT-qPCR	Signal transduction; Lipid transport; cell wall remodelling; stress response	Xiong et al., 2022
		*In vitro*	RT-qPCR		Li et al., 2015
	*Oriza sativa*	*In vitro*	RT-qPCR		Xu et al., 2022
	*Triticum aestivum*	*In planta*	RT-qPCR		Kouidri et al., 2018
Fasciclin-like arabinogalactan proteins	*Arabidopsis thaliana*	*In planta*	SDS-PAGE; Immunoblot; Protein blots	Cell wall formation; cell expansion; signalling; stress responses	Ma et al., 2023
		*In vitro*	MALDI-TOF MS; RNA Gel-Blot		Johnson et al., 2003
	*Areca catechu*	*In vitro*	RT-qPCR; Fluorescence microscopy		Li et al., 2023
	*Olea europaea*	*In vitro*	SDS-PAGE; LC-MS/MS		Pires et al., 2025b
Feronia	*Arabidopsis thaliana*	*In vitro*	RT-qPCR; Immunoblot	Plant growth and reproduction; morphogenesis; hormone signalling; immunity; stress responses	Wang et al., 2024
		*In planta*	RT-qPCR; Immunoblot; Confocal microscopy		Lin et al., 2022
		*In planta*	RT-qPCR; Western blot		Wang et al., 2020
	*Oriza sativa*	*In vitro*	NMR; Microscopy		Wong et al., 2007
Heat shock proteins	*Solanum lycopersicum*	*In planta*	SDS-PAGE; HPLC	Response to stress; cellular survival; cell signalling; immunity	Alcântara et al., 2025
	*Olea europaea*	*In vitro*	SDS-PAGE; LC-MS/MS		Pires et al., 2025b
	*Sorghum bicolor*	*In vitro*	iTRAQ, LC-MS/MS		Ngwenya et al., 2024
					Ngcala et al., 2020;
Receptor-like kinases	*Olea europaea*	*In vitro*	SDS-PAGE; LC-MS/MS	Response to abiotic stress; phytohormone signaling; immunity; plant development and reproduction	Pires et al., 2025b
	*Triticum aestivum*	*In planta*	RT-qPCR		Yan et al., 2023
	*Arabidopsis thaliana*	*In vitro*	RT-qPCR; Western blot		Wang et al., 2020
		*In planta*	RT-qPCR		Li et al., 2019
	*Gossypium* spp.	*In planta*	RT-qPCR		Sun et al., 2018
Penetration 1	*Arabidopsis thaliana*	*In planta*	Confocal microscopy	Targeted secretion in response to pathogens; growth and development	Liu et al., 2022
		*In planta*	LC-MS/MS		Johansson et al., 2014
		*In planta*	SDS-PAGE; Immunoblot		Assaad et al., 2004
Tetraspanin	*Arabidopsis thaliana*	*In planta*	Immunoblot; qRT−PCR	Marker of EV subpopulations; stress-responsive secretion; plant immunity	Koch et al., 2025
	*Solanum betaceum*	*In vitro*	RT-qPCR		César, 2023
	*Salvia dominica*	*In vitro*	SDS-PAGE; Western blot		Boccia et al., 2022

HPLC, High-performance liquid chromatography; iTRAQ, Isobaric tags for relative and absolute quantification; LC, Liquid chromatography; MALDI-TOF, Matrix-Assisted Laser Desorption/Ionization Time-of-Flight; MS/MS, Tandem mass spectrometry; qRT−PCR, Quantitative real-time reverse transcription PCR; SDS-PAGE, Sodium dodecyl sulfate-polyacrylamide gel electrophoresis.

### 5.1 Extraction and purification methods of extracellular vesicles

Efficient recovery of plant EVs is essential to preserve their structural integrity, stability and biological activity. While for the isolation of EVs in mammalian systems several methods have been developed, as a consequence of the extensive investigation that has been carried out, those from plants remain understudied, largely due to a lack of standardized protocols (Maricchiolo et al., 2026). Nevertheless, with the growing interest in plant EVs research, several approaches have been proposed to improve the specificity and efficiency of extraction. Compared to animals, the isolation of plant-derived vesicles requires additional considerations due to the complex composition of plant matrices, such as the plant cell wall. In plants, EVs extraction generally relies on juice, flesh, roots, seeds, dried tissues, or CM (Li et al., 2026). Because plant EVs are predominantly located in the apoplastic space, the isolation represents the most critical step in their extraction. Consequently, the choice of extraction method depends on the specific goals of the study—including the desired purity, yield and the type of sample analysed (Ye et al., 2025).

The methods used for plant EVs isolation are largely based on these mammalian protocols, with the additional step of collecting APF. In cell culture systems, EVs are commonly isolated from CM, where they accumulate as a result of cellular secretion (Zhang et al., 2020). Because plant EVs accumulate either in the apoplast or in CM, the isolation of a clean extracellular medium is critical for downstream analyses (Rutter and Innes, 2017; Huang et al., 2022). Current isolation methods generally rely on vesicle size, density, or specific surface markers, with the most commonly used approaches including differential ultracentrifugation and ultrafiltration, which exploit size- and density-dependent separation principles (Nueraihemaiti et al., 2025).

Differential ultracentrifugation is the most widely used method for isolating EVs from cell culture supernatants and biological fluids. In plant systems, once APF has been collected, vesicles are typically concentrated through sequential centrifugation steps in which progressively higher centrifugal forces remove contaminants of decreasing size, culminating in ultracentrifugation to sediment small vesicles (Huang et al., 2025). Although this approach is relatively straightforward and does not require specialised reagents or costly consumables, the mechanical stress generated during centrifugation and handling may compromise vesicle integrity (Doyle and Wang, 2019). In addition, the performance of this technique is influenced by several experimental parameters, including rotor configuration, centrifugal force, duration and temperature, as well as sample-related factors such as viscosity and dilution, which can ultimately affect separation efficiency and vesicle recovery. Despite its widespread use, differences in centrifugation parameters and APF collection procedures mean that a universally standardised protocol for plant EVs isolation is still lacking (Huang et al., 2021). To increase vesicle purity, differential ultracentrifugation is frequently coupled with complementary purification methods such as density gradient ultracentrifugation, ultrafiltration or size-exclusion chromatography (Li et al., 2026). Density gradient ultracentrifugation separates vesicles according to their resilient density using sucrose or iodixanol gradients, improving sample purity and vesicle integrity, although it is relatively time-consuming and requires specialised equipment (Maricchiolo et al., 2026). Ultrafiltration relies on membranes with defined pore sizes to separate particles according to size, including nanomembranes ranging from approximately 20 to 200 nm (Doyle and Wang, 2019) or membranes with specific molecular weight cut-offs that enable the selective capture of vesicles from cell culture supernatants or biological fluids (Maricchiolo et al., 2026); however, membrane obstruction and reduced vesicle recovery have been reported as major limitations (Palencia et al., 2025).

The isolation of plant EVs systems remains a technically challenging task due to the need to preserve vesicle integrity while achieving sufficient purity and yield. Differential ultracentrifugation and ultrafiltration are among the most widely used methods, each with distinct advantages and limitations, and their combination is often employed to optimise recovery. The choice of approach must consider the specific characteristics of the biological matrix, the target vesicle population, and the intended downstream applications. Continued methodological refinement will be essential to fully exploit the potential of EVs in both basic research and biotechnological applications, particularly focused on SE.

### 5.2 Extracellular vesicles as potential mediators of somatic embryogenesis signalling

EVs, as previously introduced, comprise a heterogeneous population of vesicles with distinct biogenesis pathways, including endosome-derived and plasma membrane-derived vesicles. However, in SE, the specific EV subtypes involved remain poorly characterised, and most evidence derives from EVs-enriched extracellular fractions rather than from purified vesicle populations.

Among the proteins identified in EVs-enriched fractions or extracellular secretomes of embryogenic cultures, only a subset has also been independently associated with SE. Fasciclin-like arabinogalactan proteins (FLAs), members of the Arabinogalactan protein (AGP) family, are among the most consistently reported extracellular proteins linked to embryogenic competence. FLAs are associated with membrane microdomains involved in vesicle trafficking and EVs biogenesis (Kouidri et al., 2018). In *O. europaea*, several FLAs were identified in the extracellular secretome of embryogenic cultures and assigned to apoplast- and vesicle-related GO categories (Pires et al., 2025b). Independent studies in embryogenic tissues further support the involvement of AGPs/FLAs in SE. In *Areca catechu*, genes encoding for FLA7–1 and FLA7–2 were co-expressed with genes related to hormone signalling and cell wall remodelling during SE induction (Li et al., 2023).

Heat shock proteins (HSP70 and HSP90) and the ER chaperone BINDING IMMUNOGLOBULIN PROTEIN (BiP) are recurrent components of plant EV proteomes and extracellular fractions (Pobre et al., 2019). In embryogenic systems, proteomic analyses based on iTRAQ and LC-MS/MS identified HSPs among secreted proteins recovered from the culture medium of *S. bicolor* embryogenic suspension cultures subjected to stress conditions (Ngcala et al., 2020; Ngwenya et al., 2024). Similarly, LC-MS/MS analysis of the CM from *O. europaea* embryogenic cultures also detected stress-related chaperones in the extracellular secretome (Pires et al., 2025b). These observations support the association of stress-responsive extracellular proteins with embryogenic cultures and cellular reprogramming during SE.

Tetraspanins, particularly TET8, are recognised markers of plant EVs due to their enrichment in apoplastic vesicles and association with vesicle trafficking (Rutter and Innes, 2017; Koch et al., 2025). In *Solanum betaceum*, embryogenic *calli* showed a higher abundance of TET8-positive vesicles compared with non-embryogenic *calli* (César, 2023), indicating that changes in vesicle dynamics may be associated with the acquisition of SE. Although the functional role of TET8 during SE remains unclear, these observations provide preliminary evidence linking canonical plant EV markers with embryogenic cultures.

Collectively, the available evidence suggests that some proteins identified in EV-enriched extracellular fractions from embryogenic cultures are also recurrently associated with SE in independent experimental systems. Nevertheless, the available studies consistently associate these proteins with biological processes known to be central to embryogenic transition, including stress responses, extracellular signalling, cell wall remodelling, and developmental reprogramming. Specifically, proteins linked to the acquisition of SE, proteostasis, and receptor-mediated signalling have been frequently found in both extracellular fractions and embryogenic tissues, indicating that EV-associated cargoes may play a role in the development and maintenance of the embryogenic state. Although the functional roles of specific EV populations during SE remain largely unresolved, current findings support the hypothesis that extracellular vesicle-mediated communication participates in the cellular reprogramming events underlying SE.

## 6 Conclusions

Diverse strategies have been explored to overcome the recalcitrance observed in SE. While many factors have been considered, an important aspect that is often overlooked but crucial to the SE response is the release of organic bioactive molecules by explants into the culture medium. During *in vitro* culture, a broad spectrum of molecules is actively released by explants. Their accumulation in the extracellular environment reflects dynamic metabolic exchanges between the explant and the culture medium, suggesting that the culture medium itself becomes a biologically active compartment rather than a passive support system. These molecules may significantly influence SE efficiency, given their involvement in essential biological processes and their potential roles in cellular communication. The current review highlights the biomolecules secreted during SE and emphasises their essential role in regulating the embryogenic process, acting as key components of the extracellular environment that mediate communication between explants and the culture medium and influence critical processes. Looking toward future perspectives, understanding how these extracellular signals operate and how they interact with intracellular pathways offers promising possibilities for improving SE efficiency. However, several research gaps remain. A major challenge lies in the standardisation of methodologies used in SE studies, including the preparation of conditioned medium, as well as the analytical approaches used in proteomic and metabolomic studies, all of which can significantly influence the comparability and interpretation of results. Additionally, extracellular vesicles represent a further methodological frontier, requiring specific consideration for their isolation and characterisation from plant tissues. Distinguishing passive leakage from active secretion in SE cultures also remains a critical limitation for correctly interpreting secretome data. Furthermore, integrating multi-omics approaches will be essential for identifying functional biomarkers associated with embryogenic competence and for establishing mechanistic models linking secreted factors with intracellular reprogramming. Altogether, these perspectives open new insights for understanding SE and provide a foundation for developing innovative strategies to overcome current challenges and broaden the applications of this important biological process.
